# Cilostazol alleviate nicotine induced cardiomyocytes hypertrophy through modulation of autophagy by CTSB/ROS/p38MAPK/JNK feedback loop

**DOI:** 10.7150/ijbs.43825

**Published:** 2020-04-27

**Authors:** Shu-ya Wang, Xi Ni, Ke-qing Hu, Fan-liang Meng, Min Li, Xiao-li Ma, Ting-ting Meng, Hui-hui Wu, Di Ge, Jing Zhao, Ying Li, Guo-hai Su

**Affiliations:** 1 Jinan Central Hospital Affiliated to Shandong University, Jinan, China.; 2 Central Hospital Affiliated to Shandong First Medical University, Jinan, China.; 3 Affiliated Hospital of Shandong University of Traditional Chinese Medicine, Jinan, China.; 4 School of Biological Science and Technology, University of Jinan, China.; 5 Development Biology, School of Life Science, Shandong University, Jinan, China.

**Keywords:** nicotine, cardiomyocytes hypertrophy, autophagy, CTSB, ROS, p38MAPK/JNK.

## Abstract

Nicotine is proved to be an important factor for cardiac hypertrophy. Autophagy is important cell recycling system involved in the regulation of cardiac hypertrophy. Cilostazol, which is often used in the management of peripheral vascular disease. However, the effects of cilostazol on nicotine induced autophagy and cardiac hypertrophy are unclear. Here, we aim to determine the role and molecular mechanism of cilostazol in alleviating nicotine-induced cardiomyocytes hypertrophy through modulating autophagy and the underlying mechanisms. Our results clarified that nicotine stimulation caused cardiomyocytes hypertrophy and autophagy flux impairment significantly in neonatal rat ventricular myocytes (NRVMs), which were evidenced by augments of LC3-II and p62 levels, and impaired autophagosomes clearance. Interestingly, cathepsin B (CTSB) activity decreased dramatically after stimulation with nicotine in NRVMs, which was crucial for substrate degradation in the late stage of autophagy process, and cilostazol could reverse this effect dramatically. Intracellular ROS levels were increased significantly after nicotine exposure. Meanwhile, p38MAPK and JNK were activated after nicotine treatment. By using ROS scavenger N-acetyl-cysteine (NAC) could reverse the effects of nicotine by down-regulation the phosphorylation of p38MAPK and JNK pathways, and pretreatment of specific inhibitors of p38MAPK and JNK could restore the autophagy impairment and cardiomyocytes hypertrophy induced by nicotine. Moreover, CTSB activity of lysosome regained after the treatment with cilostazol. Cilostazol also inhibited the ROS accumulation and the activation of p38MAPK and JNK, which providing novel connection between lysosome CTSB and ROS/p38MAPK/JNK related oxidative stress pathway. This is the first demonstration that cilostazol could alleviate nicotine induced cardiomyocytes hypertrophy through restoration of autophagy flux by activation of CTSB and inhibiting ROS/p38/JNK pathway, exhibiting a feedback loop on regulation of autophagy and cardiomyocytes hypertrophy.

## Introduction

Nicotine is considered as the most addictive component of cigarette, which attracted extensive attention since its important function in the cardiovascular system. Recently, several studies have reported that nicotine stimulus caused cardiac hypertrophy in vitro and in vivo experiments 1. However, the molecular mechanism of nicotine induced cardiac hypertrophy is not fully understood yet. Cardiac hypertrophy is a maladaptive response to cardiac insults, such as hypertension, myocardial infarction, external adverse stimulus. Initially, cardiac hypertrophy may serve as a compensatory response. However, sustained or excessive hypertrophic responses leads to left ventricular dilation, contractile dysfunction, and ultimate heart failure. Demonstrating the molecular mechanisms of left ventricular hypertrophy, may suggest novel therapeutic strategies. In the present study, we will investigate the effects and molecular mechanisms of nicotine induced cardiomyocytes hypertrophy.

Autophagy is a highly conserved catabolic cellular process for protein and nutrient recycling. During autophagy, an isolation membrane sequesters long-lived proteins and organelles that are damaged or malfunctioning to form the autophagosome, then the autophagosome fuses with the lysosome to become an autolysosome and degrades the materials contained within it. Dysregulated autophagy can cause various cardiovascular diseases including hypertrophy, heart failure and ischemic heart diseases 2. Recently, it has been reported that autophagy inhibition or autophagy flux impairment can cause cardiac hypertrophy 3-6. Enhanced autophagy may attenuate cardiac hypertrophy 7-9. In bronchial epithelial cells, nicotine was reported to be involved in the regulation of autophagy and emphysema progression 10, 11. However, in heart, the role of nicotine on autophagy and whether nicotine induces cardiac hypertrophy through autophagy impairment are unknown. We wondered the effects of nicotine on autophagy and hypertrophy in NRVMs and the corresponding mechanisms.

Cilostazol, as a selective inhibitor of phosphodiesterase-3, has been known for increasing intracellular cAMP levels 12, 13. While Bai Y et.al reported that cilostazol can attenuate superoxide production and reduce the infarct size in rabbit by activating CTSB 14. Furthermore, cilostazol has been identified to protect rat from Parkinson's disease through enhancing autophagy 15. So we speculated that cilostazol could relieve the nicotine-induced autophagy impairment through affecting the CTSB. CTSB, member of lysosomal proteases family, which consists of 11 members, is approximately 43 kb in size, responsible for the degradation of intracellular and extracellular proteins in the late stage of autophagy^16^, ^17^. Yong Jiang et.al reported that CTSB mediated degradation of Dab2 and induced autophagy 18. Recent studies demonstrated that CTSB participate in many pathological processes, such as angiogenesis, obesity, diabetes and cancer 19-21. However, the variation of CTSB activity, and the detailed role and mechanism of CTSB in autophagy impairment mediated cardiomyocytes hypertrophy remain unclear.

Oxidative stress is mainly related to the imbalance of oxidation and anti-oxidation in the body or cells, resulting in the accumulation of oxides. Reactive oxygen species (ROS) is one of the main intermediate oxide, which plays a vital role in various cardiovascular diseases, such as hypertension, ischemic heart disease, atherosclerosis, and cardiac hypertrophy or heart failure 22. ROS can be cleared up by autophagy under physiological conditions. It has been reported that pathological accumulation ROS can impaired autophagy flux, in turn, and ROS scavenger can reverse this effect 23, 24. Although Bodas M et.al reported that nicotine can induce the apoptosis and senescence through autophagy flux impairment in bronchial epithelial cell 10, 25, 26, the role of nicotine plays on NRVMs remains unclear. Whether nicotine induces cardiomyocytes hypertrophy in ROS-dependent autophagy flux impairment and induced the decreased cathepsin enzyme activity of lysosome has not been reported yet.

Mitogen -activated protein kinase (MAPK) is a group of serine-threonine protein kinases that can be activated by different extracellular stimuli, such as cytokines, neurotransmitters, hormones, cell stress and cell adhesion, which conventionally consists of ERK, JNK, and p38 27-29. Both JNK, also known as stress-activated protein kinases (SAPKs), and p38 signaling, as the downstream of ROS, participate in the modulation of autophagy, which attract widely attention in recent studies. Activated JNK has been reported can stimulate autophagy and induce the formation of autophagosome, while its sustained activation will block the fusion of autophagosome and lysosome 25, 30-32. P38 signaling is well known for its pro-apoptosis effect in most cases 30, 33, 34. Yingli He et.al reported that LPS inhibited autophagy initiation and flux through phosphorylation of p38 and inhibiting the binding of ULK1 and ATG13 35.However, whether p38 involved in the regulation of late stage of autophagy influx hasn't been reported. In the present study, we wonder whether p38 could also interrupt the fusion of autolysosome and induce the autophagy flux impairment through ROS-dependent activation. And this new sight of mechanism will provide a brand new point for modulating autophagy.

The aims of the present study were to determine the effects of nicotine and cilostazol in cardiomyocytes hypertrophy and autophagy flux, and explore the role of CTSB in these processes and the potential relationship between CTSB and nicotine-induced cardiomyocytes hypertrophy through modulating autophagy in NRVMs.

## Materials and methods

### Reagents

Antibodies of LC3 (#12741), phosphor-p38 MAPK (Thr180/Tyr182, #4511), p38MAPK (#8690), phosphor-JNK (Thr183/Tyr185, #4668), JNK (#9252) were obtained from Cell Signaling Technology. Antibody of p62 (PM045) was obtained from Medical & Biological Laboratories Co., Ltd. Antibodies of LAMP1 (ab24170) and LAMP2 (ab25631) were obtained from abcam. Antibody of CTSB (12216-1-AP) was obtained from Proteintech. Antibody of pro-CTSB (6679) was obtained from BioVison. SP600125, SB203580, N-acetyl cysteine were purchased from Sigma-Aldrich. Cilostazol was purchased from Selleck.

### Cell culture

#### Primary culture of NRVMs and adenovirus transfection

NRVMs were prepared as previously described 36. Briefly, 1- to 3-day-old wistar rats were anesthetized with isoflurane and ventricles were minced and digested in phosphate buffered saline (PBS) containing 200 U type II collagenase and 0.4% horse serum for three cycles. The cells were then centrifuged and suspended in Dulbecco's modified Eagle's medium containing 5% fetal bovine serum and 8% horse serum. A single 1.5 h pre-plating step was used to further remove non-cardiomyocytes. Non-cardiomyocytes attached readily to the bottom of culture dishes. The unattached myocytes were plated at 1×10^5^ cells/ml in the same medium as above and supplemented with 0.1 mM 5-Bromo-2-deoxyUridine (BrdU). Cells were placed in a serum-free medium for 24 h before experiments. NRVMs identity was confirmed by morphological examination and by staining with an anti-sarcomerica-actin antibody. Most (> 95%) of the cells were identified as NRVMs. For adenovirus transfection, NRVMs were infected with recombinant adenovirus coding for RFP-GFP-LC3 (2×10^10^ PFU/ml in stock solution, 1: 5,000 dilution) and incubated for 48 h before experiments.

#### Hematoxylin-eosin staining (HE staining)

HE staining was performed to quantify the area of cardiomyocytes. After the culture of cardiomyocytes, nicotine or vehicle was added to treat cells for 48h, 4% paraformaldehyde was used to fix cells for 10 min, washed with PBS for 1 min and repeated 3 times. Hematoxylin and eosin (H&E) staining was performed. Dehydrating using graded ethanol, vitrification by dimethylbenzene and mounting with neutral gum. Photographs were obtained by microscope.

### Real-time polymerase chain reaction

RT-PCR was performed as previously described 36. The PCR primers sequences used in this study were as follows: β-actin, 5'-CGTTGACATCCGTAAAGACC-3' (forward) and 5'-TAGAGCCACCAATCCACACA-3' (reverse); BNP, 5'-GCTGCTTTGGGCAGAAGATA-3' (forward) and 5'-GGAGTCTGCAGCCAGGAGGT-3' (reverse); ANP, 5'-GGGGGTAGGATTGACAGGAT-3' (forward) and 5'-GGATCTTTTGCGATCTGCTC-3' (reverse); β-MHC, 5'-CGCTCAGTCATGGCGGAT-3' (forward) and 5'-GCCCCAAATGCAGCCAT-3' (reverse); CTSB, 5'-GTTGGGTTCAGCGAGGACAT-3' (forward) and 5'-CCAAATGCCCAACAAGAGCC -3' (reverse). The PCR reactions were performed using the following conditions: denaturation at 95 ℃ (5 min) and 45 cycles for β-actin, β-MHC, BNP, and ANP consisting of 95 ℃ (10 s), 60 ℃ (20 s), and 72 ℃ (30 s). The relative level of each mRNA was normalized to that of β-actin.

### Transmission electron microscope (TEM)

NRVMs were treated with nicotine or PBS for 6h. Cells in both groups were washed with PBS for three times. Collected cells with scraper into 1.5 ml tubes, then centrifuged for 3min at 5000 rpm. Discarded PBS and fixed with glutaraldehyde. The samples were sent to transmission electron microscopy. Images were captured with corresponding system.

### Magic Red Cathepsin assay

NRVMs were seeded into 96-plate with black walls and a clear bottom. Treated cells with nicotine in different dose (1 nM, 10 nM, 100 nM, 1 μM, 10 μM, 100 μM, 500 μM) or PBS for 12h, Baf A1 or rapamycin (Rap) was used as controls. PP242, which could activate enzyme activity of cathepsin B, was used as a positive control. NAC, SP600125 and SB203580 were also used to further test the hypothesis. Add 50 μL DMSO into both CTSB assay to make the 260× stock solution, then 450 μL diH_2_O was added to dilute the solution to 26× staining solution. Incubate the cells with culture medium with staining solution for 45 min at 37°C. After washing with PBS for 3 times, analysis the red fluorescence intensity using fluorescence plate reader with setting the optimal excitation and emission wavelength tandem of 592 nm and 628 nm, respectively.

### Western blotting analysis

Western blotting and quantification of the abundance of relative proteins were performed as described previously 37. Briefly, cells were lysed in protein lysis buffer (1% SDS, 25 mM Tris-HCl (pH 7.5), 4 mM EDTA, 100 mM NaCl, 1 mM PMSF, 10 mg/ml leupeptin and 10 mg/ml soybean trypsin inhibitor). The protein concentration of the lysates was determined using the Coomassie Brilliant Blue protein assay. Protein extracts (10 - 20 μg) were loaded on 12% SDS polyacrylamide gels, subjected to electrophoresis, and transferred to a PVDF membrane. The membranes were incubated with anti-Lamp2, anti-LC3, anti-p62, anti-p-p38MAPK (Thr180/Tyr182), anti-p38MAPK, anti-p-JNK (Thr183/Tyr185), anti-JNK, anti-CTSB, anti-pro-CTSB or anti-β-actin antibodies (1:1,000 dilution). The indicated proteins were detected with a horseradish peroxidase-conjugated IgG. The band intensity was quantified using Quantity One software (Bio-Rad, USA) and normalized to β-actin levels.

### Intracellular ROS detection

Following nicotine treatment, intracellular ROS was detected by fluorescence microscope using dichlorofluorescein diacetate (DCFH-DA) staining. Briefly, the NRVMs were incubated with 5 mM DCFH-DA for 30 min at 37 ℃ protecting from light and then were washed with serum-free medium for three times. The fluorescence was excited at the wavelength of 485 nm and the corresponding emission wavelength was 525 nm.

### Statistical analysis

The data are presented as the mean ± SEM. The statistical analysis of differences between two groups were assessed with the unpaired t-test, and the differences among more than three groups were assessed by one-way analysis of variation (ANOVA) followed by a Bonferroni's tests for post hoc analysis and multiple comparison tests with Prism Software version 7.0 GraphPad Software, San Diego California USA). The figures were processed with Adobe Photoshop software. The mean values were derived from at least three independent experiments. Differences with a *p* < 0.05 were considered statistically significant.

## Results

### Nicotine stimulation induced autophagy flux impairment and cardiomyocytes hypertrophy in NRVMs

**Figure 1:**
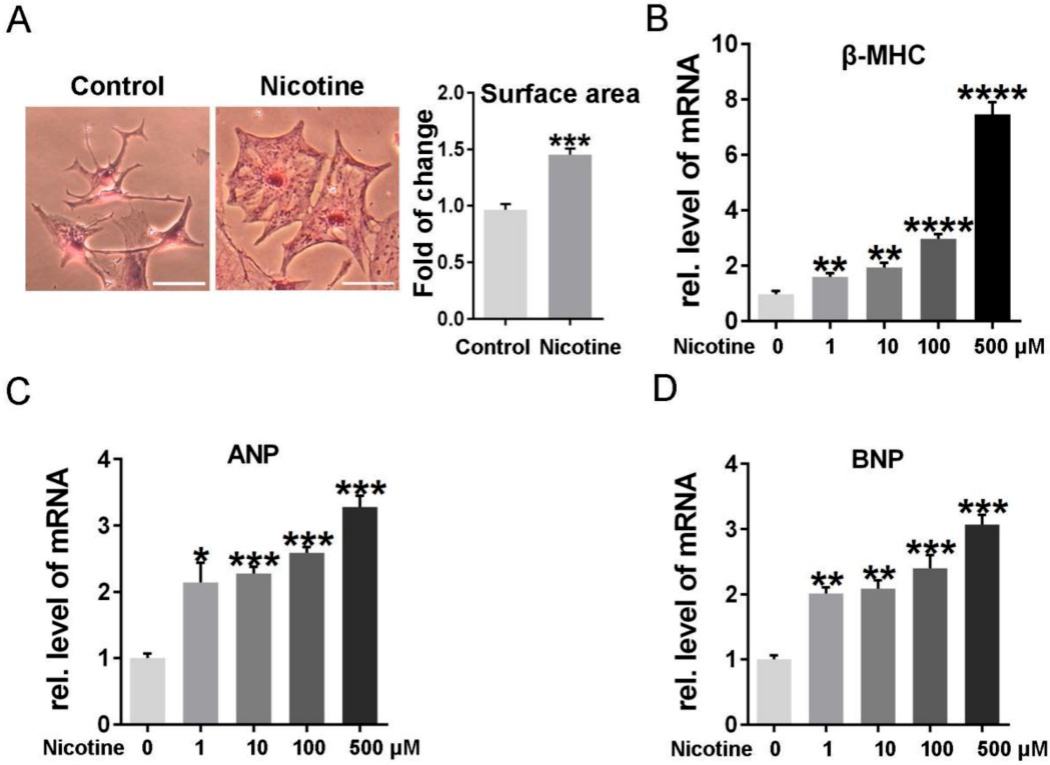
** Different concentrations of nicotine treatment caused cardiomyocytes hypertrophy significantly. (A)** HE staining was performed to detect the cell area after stimulation with nicotine, and quantification was analyzed by Image J software. (Scale bar = 20μm) qPCR was performed to determine the cardiac hypertrophy markers, **(B)** β-MHC, **(C)** ANP and **(D)** BNP. (****, p < 0.0001; ***, p < 0.001; **, p < 0.01; *, p < 0.05, n = 3).

**Figure 2:**
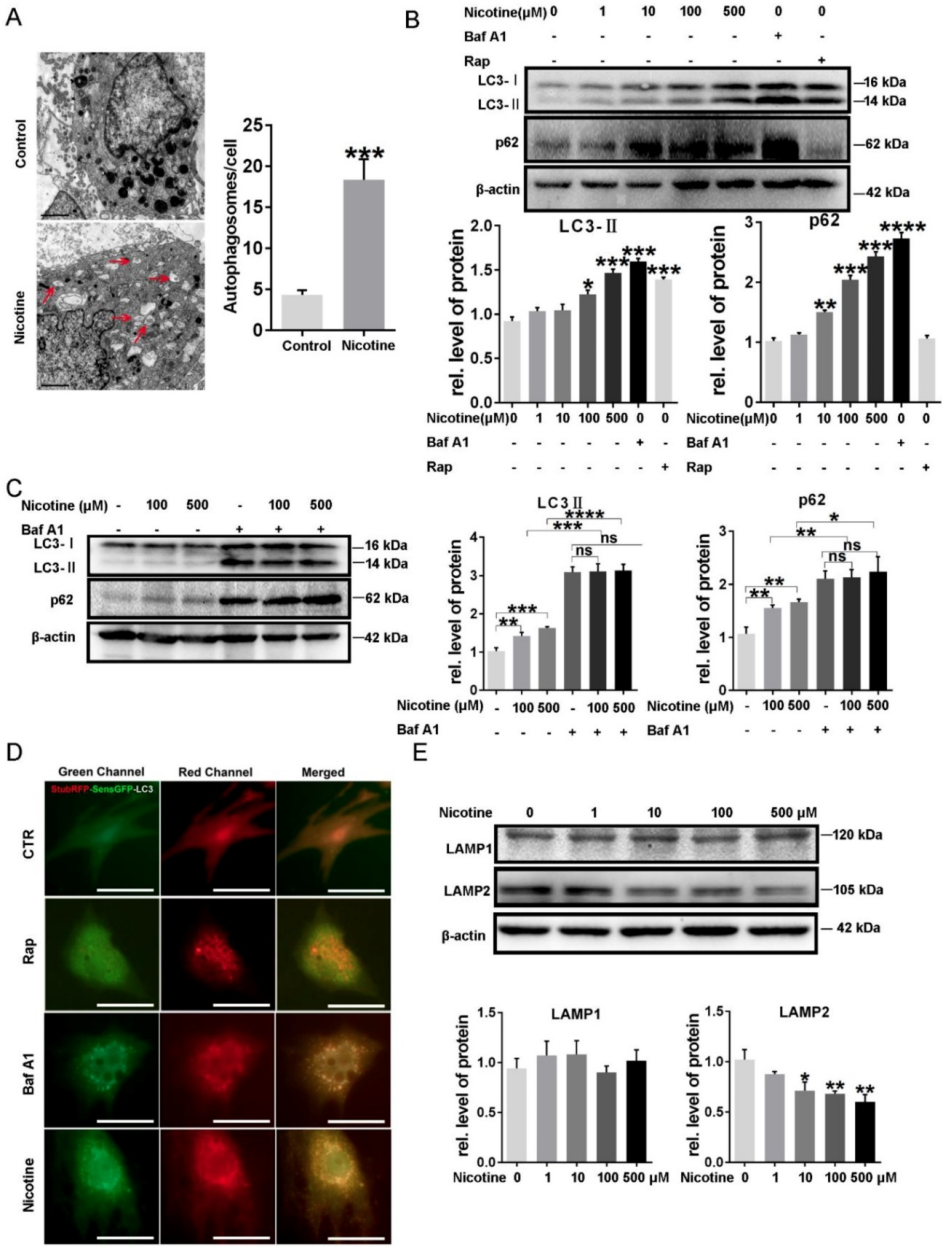
** Nicotine induced autophagy impairment in NRVMs. (A)** Transmission electron microscope (TEM) was used to determine the effect of nicotine on autophagy flux, and (Scale bar=2 μm) **(B)** Western blot was also performed to determine the autophagy marker LC3-II and its specific substrate p62 expression, BafA1 (100 nM) and Rap (10 μM) were used as negative and positive control respectively. (C) The effects on autophagy flux after combined treatment of nicotine with bafA1. **(D)** ADV-RFP-GFP-LC3 transfection was used to detect the nicotine-induced autophagy impairment. Representative immunofluorescence images of NRVMs expressing RFP-GFP-LC3 and treated with nicotine (100 μM), Rap, BafA1 or vehicle control for 24 hours. Representative of n = 3 experiments. (Scale bar, 20 μm) **(E)**The key determinant of autophagosome-lysosome fusion LAMP2 and lysosome marker LAMP1 were tested by Western blot. (****, p < 0.0001;***, p < 0.001; **, p < 0.01; *, p < 0.05 n = 3).

To determine the effects of nicotine on cardiomyocytes hypertrophy, NRVMs were stimulated with 1, 10, 100, 500 μM nicotine for 48 h. As shown in figure 1, the cardiomyocytes surface area (Figure 1A) and cardiac hypertrophy marker, ANP, BNP and β-MHC expression were significantly increased after treatment with nicotine (Figure 1B-D). To investigate whether nicotine induced autophagy impairment in cardiomyocytes, the morphological changes of autophagosomes were observed by transmission electron microscopy (Figure 2A). A large number of autophagosomes were observed after the nicotine treatment compared to the control group, and the black dots in the control group were lysosomes. The conjugation of the soluble form of LC3 (LC3-I) with phosphatidylethanolamine and conversion to a non-soluble autophagosome associated form (LC3-II) has been generally considered as a useful sign of autophagy. Thus, we determined the expression of LC3-II. Bafilomycin A1 (BafA1) and rapamycin (Rap) were used as positive controls. After stimulation with different concentrations of nicotine, LC3-II levels were markedly increased (Figure 2B). However, the elevated level of LC3-II due to activation of autophagy or blockade of autophagy-lysosomes fusion needed further detection. Thus, we next examined the expression of p62, which is a selective substrate of autophagy. As shown in figure 2B, stimulation with nicotine caused significantly increase in p62, indicating that impaired autophagy flux in NRVMs. Moreover, we determined the LC3-II and p62 levels after combined treatment with bafA1 and nicotine or nicotine alone in NRVMs. The results demonstrated that Baf A1 caused significant increase of LC3-II and p62 in NRVMs. Stimulation of nicotine combined with Baf A1 has no significant difference versus BafA1 groups (Figure 2C). These results suggest that nicotine impaired autophagy flux may through blocking the late stage of autophagosome degradation.

To further investigate whether the stage that autophagosomes fuse with the lysosomes and form of normal autolysosomes is blocked, the relative abundance of autophagosomes and autolysosomes were assessed with adenovirus mediated transfection of RFP-GFP tandem-tagged LC3 (Figure 2D). Induction of autophagy leads to punctuate localization of LC3 on autophagosomes, which demonstrate both red and green fluorescence, with subsequent loss of green fluorescent signal on autophagosome-lysosome fusion and formation of normal autolysosomes, due to instability of GFP in the acidic intra-lysosomal environment 24. As shown in Figure 2D, NRVMs of control groups demonstrate a preponderance of autolysosomes (punctate dots that fluoresce only red, and a few autophagosomes punctate dots that fluoresce green and red, i.e. yellow). Treatment with rapamycin stimulates autophagy with enhanced autophagy flux, as evidenced by markedly increased abundance of autolysosomes without a discernible accumulation of autophagosomes, indicating intact flux. Nicotine stimulation resulted in accumulation of autophagosomes and near absence of autolysosomes which was similar to treatment with Baf A1, while the rapamycin-treated group showed few green and yellow punctate. Collectively, these data indicated that nicotine stimulation lead to impaired autophagy flux in NRVMs. LAMP2, a critical determinant of autophagosome-lysosome fusion 38, were significantly decreased after treatment of nicotine, while LAMP1, which closely tracks lysosome numbers, kept unchanged (Figure 2E). These results demonstrated the nicotine impaired autophagy flux in NRVMs.

### Cilostazol alleviate nicotine induced cardiomyocytes hypertrophy through restoration of autophagy flux by activation of CTSB

**Figure 3:**
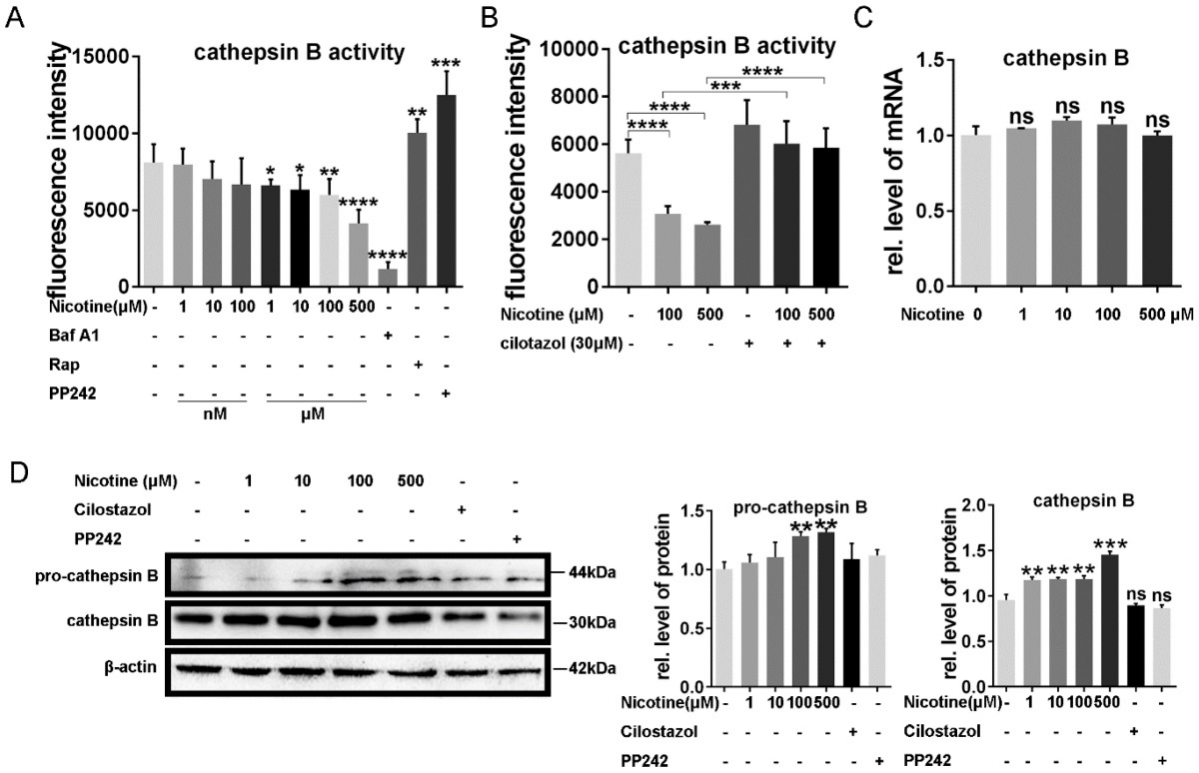
** Nicotine modulate the CTSB enzyme activity of lysosome through autophagy impairment, which reversed by cilostazol.** (A) Nicotine treatment decreased the enzyme activity of CTSB in dose-dependent manner, Rap, PP242 and Baf A1 were chosen as controls, and cilostazol could relieve this effect** (B).** mRNA level of CTSB kept unchanged after the nicotine treatment **(C)**, while the protein level of CTSB and**,** pro-CTSB showed increase under nicotine stimulation **(D).** (****, p < 0.0001; ***, p < 0.001; **, p < 0.01; *, p < 0.05, n = 3).

**Figure 4:**
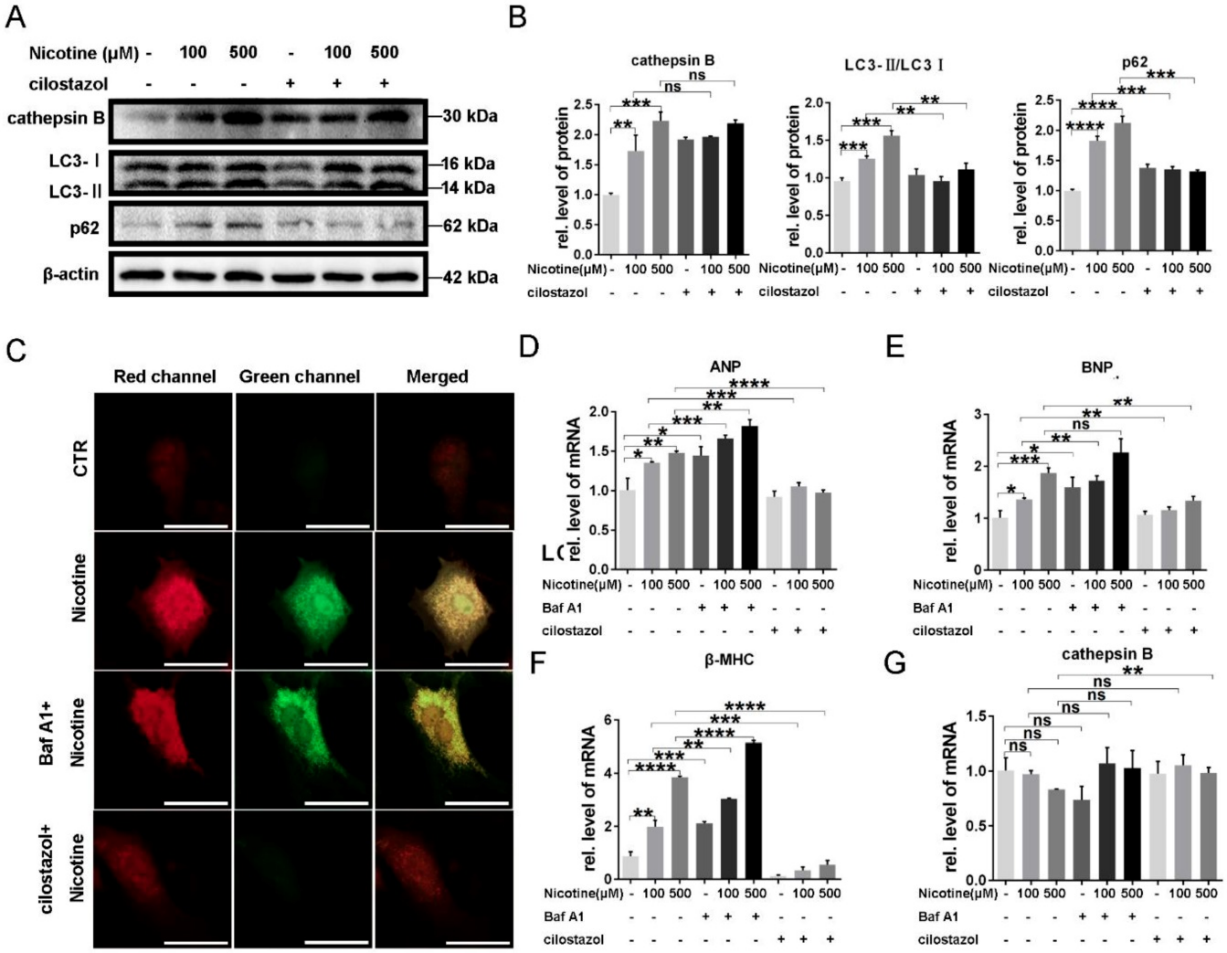
** Cilostazol relieved cardiomyocytes hypertrophy through improving autophagy flow.** Pre-treatment of cilostazol markedly decreased the marker of autophagy flux, which shown by western blot **(A)**, while the protein level of CTSB kept unchanged. Quantitative analysis was performed by Graphpad prism **(B)**. RFP-GFP-LC3 transfection was also used to test the effect of cilostazol **(C)** (Scale bar=20 μm). Cardiac hypertrophy marker, **(D-F)** ANP, BNP, β-MHC and (G) cathepsin B level were determined by qPCR (****, p < 0.0001; ***, p < 0.001; **, p < 0.01; *, p < 0.05, n = 3).

It has been reported that autophagy flux impairment plays a vital role in cardiac hypertrophy 39, 40. To further explore the inhibitory effect of nicotine on autophagy, we next test the enzyme's activity in lysosome, which was often ignored in the previous studies. CTSB ubiquitously existing in lysosome and are the most common enzymes. So we first analyzed the enzymes activity after the treatment of nicotine in different concentration for 12 h. As shown in Figure 3A, CTSB activity was inhibited significantly after simulation with nicotine, Baf A1. PP242, which could activate the enzyme activity of CTSB, was used as positive control 41, 42. As shown in figure 3A, CTSB activity was down-regulated by nicotine in dose-dependent way, while cilostazol act as an activator of CTSB rescued this effect dramatically (Figure 3B). So we wonder whether nicotine affect CTSB activity through decreasing its abundance. qPCR result demonstrated that nicotine had no effect on CTSB's mRNA level at different concentration (Figure 3C). Unexpectedly, western blot showed significantly increase of CTSB upon nicotine treatment in a dose-dependent way. While treated with nicotine at higher concentration, the protein level of pro-CTSB was also increased. Pre-treatment of cilostazol or PP242 slightly decreased the protein abundance of CTSB, but had no statistical difference versus control group (Figure 3D). Thus, nicotine involved in the regulation of autophagy influx may through decreased activity of CTSB, not its abundance. By pretreatment with cilostazol, autophagy marker LC3-II and the substrate p62 were significantly decreased, but the protein level of CTSB kept unchanged (Figure 4A-B). At the same time, transfection with recombinant adenovirus coding for RFP-GFP-LC3 showed that cilostazol could ameliorate nicotine induced impaired autophagy influx, while, Baf A1, can aggregate the accumulation of green and yellow fluorescence (Figure 4C). Next, we detected the effects of nicotine and cilostazol induced autophagy variation on cardiomyocytes hypertrophy by testing the RNA levels of ANP, BNP and β-MHC. Our results showed that the autophagy inhibitor bafA1 could aggravate nicotine caused cardiomyocytes hypertrophy, while cilostazol, which can restore autophagy flux, alleviated nicotine induced cardiomyocytes hypertrophy significantly (Figure 4D-F). However, mRNA levels of CTSB were not changed under stimulation of nicotine at 100 μM (Figure 4G). Collectively, these results demonstrated that cilostazol could alleviate nicotine induced cardiomyocytes hypertrophy mainly through restored autophagy flux mainly by activation of CTSB, not its abundance.

### Excess ROS generation contributed to nicotine induced autophagy impairment and cardiomyocytes hypertrophy and cilostazol decreased ROS level elevated by nicotine

**Figure 5:**
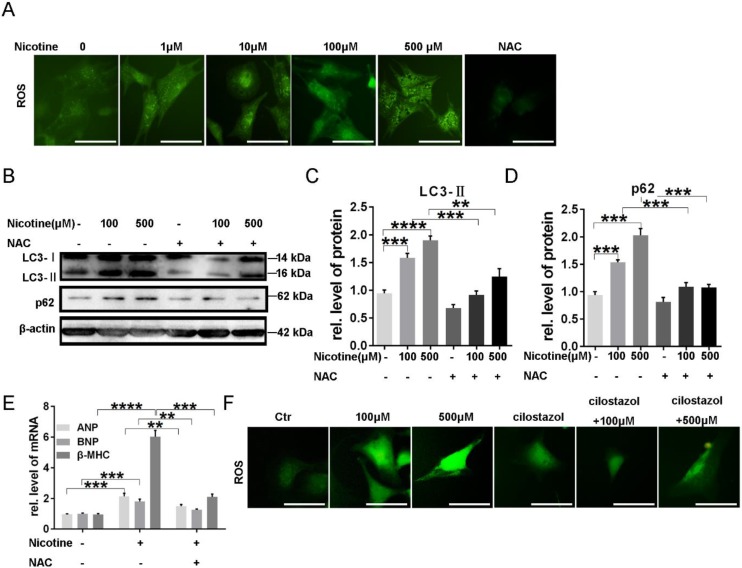
** Excess ROS production contributed to nicotine induced autophagy impairment and cardiomyocytes hypertrophy through activation of p38MAPK and JNK and cilostazol decreased ROS level elevated by nicotine. (A)** ROS levels were determined by using DCFH-DA probe, NAC decreased ROS significantly as positive control (Scale bar=20μm). **(B)** NAC pretreatment inhibited effects of nicotine on autophagy. Quantitative analysis of **(C)** LC3-II and **(D)** p62 in figure B. **(E)** Real-time PCR-based quantitation of cardiac hypertrophy markers. **(F)** Cilostazol pretreatment inhibited nicotine induced ROS accumulation (Scale bar=20 μm) (****, p < 0.0001; ***, p < 0.001; **, p < 0.01; *, p < 0.05, n = 3).

To investigate whether reactive oxygen species (ROS) plays a role in the induction of autophagy impairment by nicotine, DCFH-DA, a fluorescent probe, was used to examine cellular ROS levels. As expected, NRVMs treated with nicotine showed a remarkable enhancement in fluorescence intensity compared to the untreated control group, and NAC was selected as an positive control, which decreased ROS accumulation obviously (Figure 5A). N-acetyl-L-cysteine (NAC) is a usual anti-oxidant. Pre-incubation with NAC (0.5 mmol/l) markedly inhibited nicotine-induced autophagy impairment. As shown in figure 5B-D, autophagy marker LC3-II and the specific substrate p62 levels were down-regulated after pretreatment with NAC in NRVMs, and meanwhile cardiomyocytes hypertrophy were alleviated as indicated by decreased levels of ANP, BNP and β-MHC expression (Figure 5E). It has been reported autophagy can eliminate accumulated ROS through chaperone-mediated autophagy 43. Thus, we next detected the role of cilostazol on elimination of excessive ROS induced by nicotine. Just as our speculation, cilostazol could reduce the ROS production stimulated by nicotine (Figure 5F). Taken together, nicotine induced overproduction of ROS contributed to cardiomyocytes hypertrophy and autophagy in NRVMs, while cilostazol could counteract the influence of nicotine.

### ROS-dependent p38MAPK and JNK activation was associated to nicotine induced autophagy impairment and cardiomyocytes hypertrophy

JNK and p38MAPK, belonging to MAPK signaling pathway, are two important effectors at the downstream of ROS and also involved in the regulation of cardiac hypertrophy and autophagy. We next examined the activation of those two kinases. As shown in supplementary Fig. S1 A-C, phosphorylation of p38MAPK and JNK levels were elevated significantly under stimulation of different concentration of nicotine, indicating the participation of those two kinases in the signal transduction of nicotine. To further determine whether these two kinases were associated with ROS levels, we examined the phosphorylation levels of JNK and p38MAPK in NRVMs pretreated with NAC. The results showed that JNK and p38MAPK activation stimulated by nicotine were inhibited by NAC, suggesting the JNK and p38MAPK activation were dependent on ROS generation (supplementary Fig. S1D-F).

**Figure 6:**
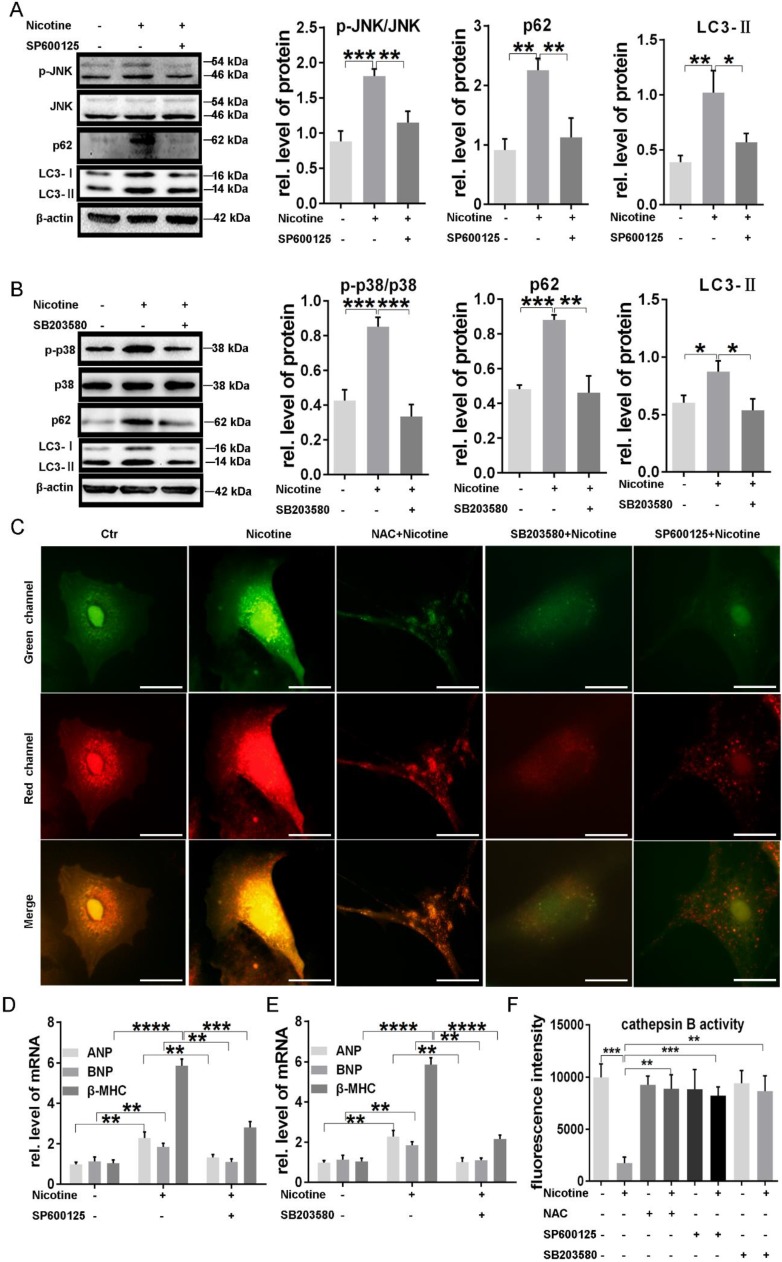
** P38MAPK and JNK were responsible for nicotine induced autophagy impairment and caused cardiomyocytes hypertrophy. (A)** JNK activation and the expression of autophagy related proteins decreased after pretreatment with the specific inhibitor of JNK SP600125. **(B)** P38MAPK phosphorylation, LC3-II and p62 protein levels were reduced after treatment with p38MAPK specific inhibitor SB203580. Autophagy flux impairment **(C)** were also reversed by NAC, SP100625 and SB203580. (Scale bar=20 μm) The specific inhibitor of **(D)** JNK and **(E)** p38MAPK pretreatment reversed nicotine induced cardiomyocytes hypertrophy and increased the CTSB activity **(F)**. (****, p < 0.0001; ***, p < 0.001; **, p < 0.01; *, p < 0.05, n = 3).

By using specific inhibitors, we further determined whether nicotine induced autophagy impairment and cardiomyocytes hypertrophy through JNK and p38MAPK activation. As shown in figure 6, pretreatment with JNK inhibitor SP600125 and p38MAPK inhibitor SB203580, could significantly recovered nicotine induced autophagy impairment in NRVMs, evidenced by decreased LC3-II and p62 levels (Figure 6A-B). ADV-RFP-GFP tandem-tagged LC3 transfection showed the effect of these inhibitors' effect on autophagy flux, and all of these three inhibitors could reverse the impaired autophagy flux (Figure 6C), which indicated the correlation between p38/JNK and autophagy flux. Furthermore, those specific inhibitors also inhibited cardiomyocytes hypertrophy caused by nicotine (Figure 6D-E). After the pretreatment of NAC, SB203580 or SP600125, nicotine induced decrease of CTSB activity was restored distinctly (Figure 6F), suggesting that the regulatory role of ROS/p38MAPK/JNK pathway on the enzymes' activity in lysosome. ROS level in NRVMs significantly declined after the pretreatment of NAC, SP600125 or SB203580, which was shown in supplementary Fig. S2.

### Cilostazol decreased the phosphorylation of p38/JNK signaling in feedback

**Figure 7:**
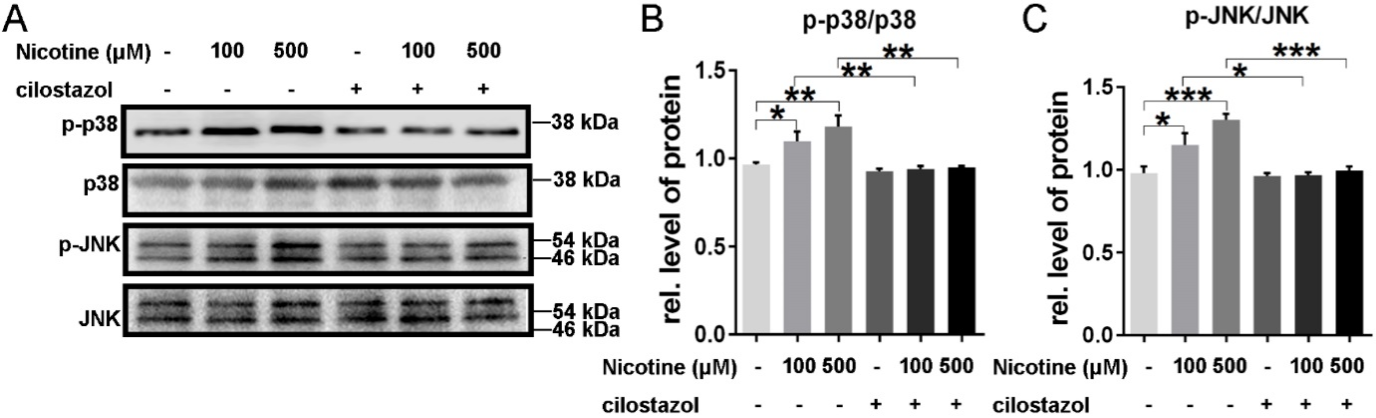
** Cilostazol depressed the activation of p38MAPK and JNK signal.** Cilostazol pre-treatment significantly inhibited the phosphorylation of p38MAPK and JNK **(A-C)**. (***, p < 0.001; **, p < 0.01; *, p < 0.05, n = 3).

Cilostazol, which could activate CTSB, was used to pretreat NRVMs. Just as our data showed, it could alleviate the nicotine-induced ROS accumulation, which reflected by the green fluorescence in figure 5F**.** And the activation of p38/JNK was also reversed by cilostazol (Figure 7A-C). These results showed that cilostazol could rescue the nicotine-induced ROS accumulation and p38/JNK phosphorylation.

## Discussion

**Figure 8:**
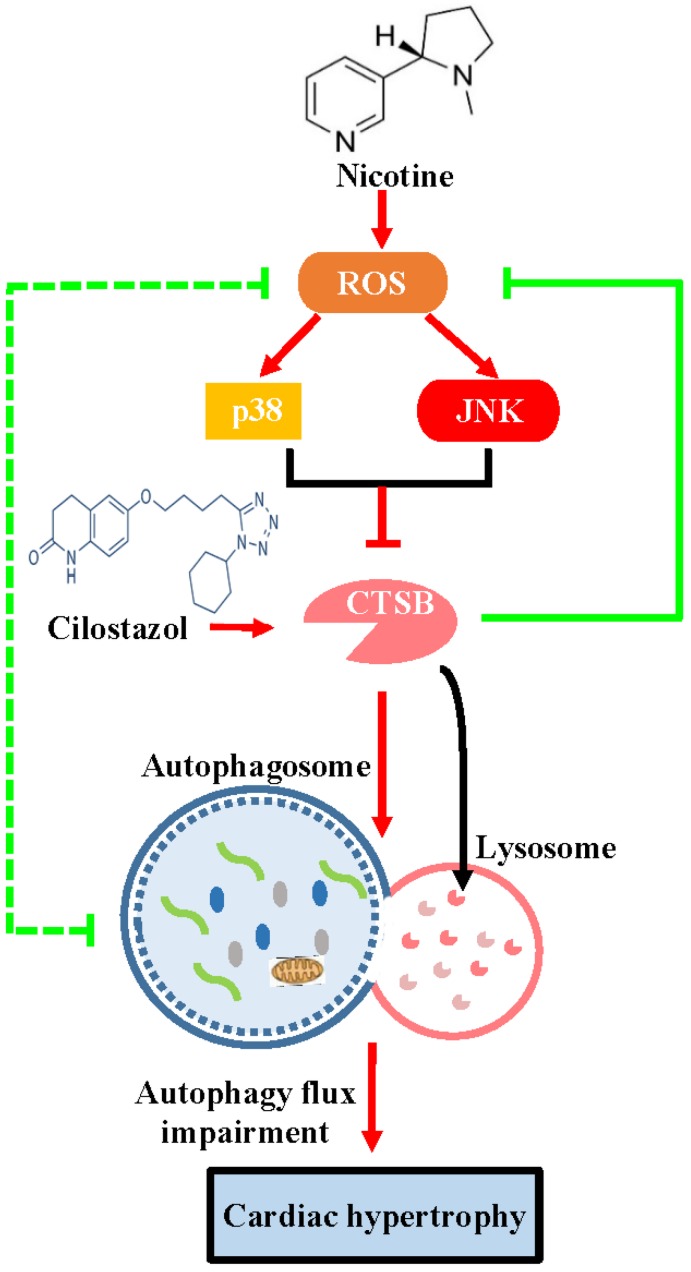
** The schematic diagram of cilostazol's modulation on nicotine-induced autophagy impairment and cardiomyocytes hypertrophy through CTSB/ROS/p38MAPK/JNK pathway.** Nicotine activates ROS/p38/JNK signaling and decreases the CTSB activity, which inhibits the fusion of autophagosome and lysosome. Just as the dashed green line showed, a negative feedback exists between ROS and autophagy flux impairment: ROS impairs the fusion of autophagosome and lysosome, and the impaired autophagy flux lead to the ROS accumulation. The nicotine-induced impaired autophagy flux leads to cardiomyocytes hypertrophy in NRVMs. Cilostazol rescues the nicotine-induced ROS accumulation, then dephosphorylates p38/JNK, alleviates autophagy impairment through stimulating CTSB activity and improves cardiomyocytes hypertrophy ultimately.

In the present study, we demonstrate for the first time that cilostazol could alleviate nicotine induced cardiomyocytes hypertrophy through restoration of autophagy flux by CTSB/ROS/p38/JNK feedback loop (Figure 8). Firstly, our results showed that nicotine, which was the most addictive component of cigarette, could induced cardiomyocytes hypertrophy and impaired autophagy flux. Nicotine has been reported to be involved in pathological mechanism of various diseases, such as emphysema, hypertension and atherosclerosis 44, 45. However, some studies have suggested existence of smoker's paradox, stating that the outcomes in smokers who have developed coronary artery disease, may be neutral or better than nonsmokers^46^. While other studies considered that smoking is associated with adverse clinical outcomes in patients undergoing revascularization with PCI or CABG 47. The existence of smoker's paradox is still controversial and potential mechanisms have not been explained. Therefore, the effects of nicotine on cardiovascular system, which is the most addictive component of cigarette, still need further investigation. Recently, the effects of nicotine on cardiac hypertrophy aroused widespread attention. However, there is very few report on this aspect. The role and molecular mechanism are still need further investigation. In the present study, we show that nicotine could induce autophagy impairment and hypertrophy in NRVMs. Upon stimulation with cilostazol, which could activate CTSB and restore autophagy flux, significantly attenuate nicotine induced cardiomyocytes hypertrophy. While treatment with bafA1, which is an autophagy flux blocker, could induce cardiomyocytes hypertrophy. These results indicated that the intact autophagy flux is crucial in avoiding hypertrophy of NRVMs.

Pathological autophagy induction is critical to the development of various diseases such as neurodegenerative disease, aging, and also cardiovascular diseases including cardiac hypertrophy. However, the molecular mechanism of autophagy flux impairment is unclear, and very few studies focused on flux of autophagy. In the present study, we showed that nicotine caused autophagy flux impairment through decreasing activity of CTSB and elevated intracellular ROS level, and subsequent activation of ROS-dependent p38MAPK and JNK pathway. Furthermore, our results showed that pretreatment with ROS scavenger NAC could improve the activity of CTSB inhibited by nicotine, and restored autophagy flux impaired by nicotine. CTSB activity decline, which probably the main direct reason of waste organelles and proteins' accumulation in ROS mediate autophagy flux impairment. These results may provide novel potential interpretation for ROS related autophagy flux impairment caused by multiple factors in some pathological conditions.

P38MAPK and JNK are two important signaling pathway at the downstream of ROS. It has been reported that p38MAPK and JNK were involved in the regulation of cardiac hypertrophy after stimulus of angiotensin II or retinoic acid 48, 49. However, whether these two signals related with nicotine induced autophagy impairment and cardiomyocytes hypertrophy is unknown. Recent study showed that JNK activation by nitrobenzoxadiazoles leads to late-stage autophagy inhibition, suggesting the role of JNK in autophagy 50. However, whether p38MAPK pathway is responsible for nicotine induced autophagy impairment is unclear yet. Our results showed that JNK as well as p38MAPK were activated after nicotine stimulation. Pretreatment with specific inhibitors of p38MAPK or JNK, could cancel the effects of nicotine on autophagy and cardiomyocytes hypertrophy as evidence by decreased expression of LC3-II and p62 as well as the cardiac hypertrophy biomarkers ANP, BNP and β-MHC. These results suggest that p38 or JNK activation was responsible for autophagy impairment in the late stage caused by nicotine, which may providing new proofs of p38 and JNK signaling pathway in clarifying the mechanism of autophagy.

Importantly, we firstly found that the activity of CTSB decreased after nicotine stimulation, while the mRNA level had no obvious change, and its relative protein level increased in concentration-dependent manner. However, someone reported that CTSB-deficiency attenuate overload-induced cardiac remodeling, which seems contradict to our result. In this report, they focused on the expression level of CTSB, not the enzyme activity. Therefore, we suspected that CTSB activity, which participates in substrate degradation at the late stage of autophagy, is more important than expression level in autophagy regulation. Cilostazol could relieve the nicotine-induced autophagy impairment and cardiomyocytes hypertrophy. Moreover, CTSB activity was also modulated by ROS generation and p38/JNK signaling in turn, pre-treatment of cilostazol alleviated the ROS stacking and p38/JNK activation. Thus, CTSB, ROS, p38/JNK formed a feedback loop, which was critical for the regulation in autophagy-related cardiomyocytes hypertrophy induced by nicotine. These findings revealed the potential link between CTSB and ROS/p38/JNK signaling. However, there are some limitations in our study. Our research focus on the nicotine effect on cardiomyocytes in vitro, more detailed evidence and animal experiments need to be explored in the future to support the conclusion.

Collectively, our results demonstrated that cilostazol could relieve nicotine induced cardiomyocytes hypertrophy through restoration of autophagy flux by CTSB/ROS/p38MAPK/JNK pathway, which may provide new sight for the molecular mechanism of autophagy and cardiomyocytes hypertrophy.

## Supplementary Material

Supplementary figures.

## References

[1] Reis Junior D, Antonio EL, de Franco MF, de Oliveira HA, Tucci PJ, Serra AJ (2016). Association of Exercise Training with Tobacco Smoking Prevents Fibrosis but has Adverse Impact on Myocardial Mechanics. Nicotine & tobacco research: official journal of the Society for Research on Nicotine and Tobacco.

[2] Hartupee J, Szalai GD, Wang W, Ma X, Diwan A, Mann DL (2017). Impaired Protein Quality Control During Left Ventricular Remodeling in Mice With Cardiac Restricted Overexpression of Tumor Necrosis Factor. Circulation Heart failure.

[3] Zhang S, Lin X, Li G, Shen X, Niu D, Lu G (2017). Knockout of Eva1a leads to rapid development of heart failure by impairing autophagy. Cell death & disease.

[4] Wu H, Wang Y, Wang X, Li R, Yin D (2017). MicroRNA-365 accelerates cardiac hypertrophy by inhibiting autophagy via the modulation of Skp2 expression. Biochemical and biophysical research communications.

[5] Zhang Y, Xu X, Ren J (2013). MTOR overactivation and interrupted autophagy flux in obese hearts: a dicey assembly?. Autophagy.

[6] Wang X, Cui T (2017). Autophagy modulation: a potential therapeutic approach in cardiac hypertrophy. Am J Physiol Heart Circ Physiol.

[7] Sureshbabu A, Syed M, Das P, Janer C, Pryhuber G, Rahman A (2016). Inhibition of Regulatory-Associated Protein of Mechanistic Target of Rapamycin Prevents Hyperoxia-Induced Lung Injury by Enhancing Autophagy and Reducing Apoptosis in Neonatal Mice. American journal of respiratory cell and molecular biology.

[8] Gu J, Hu W, Song ZP, Chen YG, Zhang DD, Wang CQ (2016). Rapamycin Inhibits Cardiac Hypertrophy by Promoting Autophagy via the MEK/ERK/Beclin-1 Pathway. Frontiers in physiology.

[9] Yan J, Yan JY, Wang YX, Ling YN, Song XD, Wang SY (2019). Spermidine-enhanced autophagic flux improves cardiac dysfunction following myocardial infarction by targeting the AMPK/mTOR signalling pathway. Br J Pharmacol.

[10] Bodas M, Van Westphal C, Carpenter-Thompson R, D KM, Vij N (2016). Nicotine exposure induces bronchial epithelial cell apoptosis and senescence via ROS mediated autophagy-impairment. Free radical biology & medicine.

[11] Bodas M, Vij N (2017). Augmenting autophagy for prognosis based intervention of COPD-pathophysiology. Respiratory research.

[12] Li J, Xiang X, Xu Z (2019). Cilostazol protects against myocardial ischemia and reperfusion injury by activating transcription factor EB (TFEB). Biotechnol Appl Biochem.

[13] Park SY, Lee HR, Lee WS, Shin HK, Kim HY, Hong KW (2016). Cilostazol Modulates Autophagic Degradation of beta-Amyloid Peptide via SIRT1-Coupled LKB1/AMPKalpha Signaling in Neuronal Cells. PloS one.

[14] Bai Y, Muqier, Murakami H, Iwasa M, Sumi S, Yamada Y (2011). Cilostazol protects the heart against ischaemia reperfusion injury in a rabbit model of myocardial infarction: focus on adenosine, nitric oxide and mitochondrial ATP-sensitive potassium channels. Clin Exp Pharmacol Physiol.

[15] Hedya SA, Safar MM, Bahgat AK (2018). Cilostazol Mediated Nurr1 and Autophagy Enhancement: Neuroprotective Activity in Rat Rotenone PD Model. Mol Neurobiol.

[16] Wu QQ, Xu M, Yuan Y, Li FF, Yang Z, Liu Y (2015). Cathepsin B deficiency attenuates cardiac remodeling in response to pressure overload via TNF-alpha/ASK1/JNK pathway. Am J Physiol Heart Circ Physiol.

[17] Man SM, Kanneganti TD (2016). Regulation of lysosomal dynamics and autophagy by CTSB/cathepsin B. Autophagy.

[18] Jiang Y, Woosley AN, Sivalingam N, Natarajan S, Howe PH (2016). Cathepsin-B-mediated cleavage of Disabled-2 regulates TGF-beta-induced autophagy. Nat Cell Biol.

[19] Zhang ZY, Mai Y, Yang H, Dong PY, Zheng XL, Yang GS (2014). CTSB promotes porcine preadipocytes differentiation by degrading fibronectin and attenuating the Wnt/beta-catenin signaling pathway. Mol Cell Biochem.

[20] Mizunoe Y, Sudo Y, Okita N, Hiraoka H, Mikami K, Narahara T (2017). Involvement of lysosomal dysfunction in autophagosome accumulation and early pathologies in adipose tissue of obese mice. Autophagy.

[21] Aggarwal N, Sloane BF (2014). Cathepsin B: multiple roles in cancer. Proteomics Clin Appl.

[22] Roy J, Galano JM, Durand T, Le Guennec JY, Lee JC (2017). Physiological role of reactive oxygen species as promoters of natural defenses. FASEB journal: official publication of the Federation of American Societies for Experimental Biology.

[23] Lee J, Giordano S, Zhang J (2012). Autophagy, mitochondria and oxidative stress: cross-talk and redox signalling. The Biochemical journal.

[24] Ma X, Liu H, Foyil SR, Godar RJ, Weinheimer CJ, Hill JA (2012). Impaired autophagosome clearance contributes to cardiomyocyte death in ischemia/reperfusion injury. Circulation.

[25] Xiang XY, Yang XC, Su J, Kang JS, Wu Y, Xue YN (2016). Inhibition of autophagic flux by ROS promotes apoptosis during DTT-induced ER/oxidative stress in HeLa cells. Oncology reports.

[26] Ou L, Lin S, Song B, Liu J, Lai R, Shao L (2017). The mechanisms of graphene-based materials-induced programmed cell death: a review of apoptosis, autophagy, and programmed necrosis. International journal of nanomedicine.

[27] Manfiolli AO, Siqueira FS, Dos Reis TF, Van Dijck P, Schrevens S, Hoefgen S (2019). Mitogen-Activated Protein Kinase Cross-Talk Interaction Modulates the Production of Melanins in Aspergillus fumigatus. mBio.

[28] Ling T, Bellin D, Vandelle E, Imanifard Z, Delledonne M (2017). Host-Mediated S-Nitrosylation Disarms the Bacterial Effector HopAI1 to Reestablish Immunity. Plant Cell.

[29] Zhang F, Zhao Q, Tian J, Chang YF, Wen X, Huang X (2018). Effective Pro-Inflammatory Induced Activity of GALT, a Conserved Antigen in A. Pleuropneumoniae, Improves the Cytokines Secretion of Macrophage via p38, ERK1/2 and JNK MAPKs Signal Pathway. Front Cell Infect Microbiol.

[30] Sui X, Kong N, Ye L, Han W, Zhou J, Zhang Q (2014). p38 and JNK MAPK pathways control the balance of apoptosis and autophagy in response to chemotherapeutic agents. Cancer letters.

[31] Lorin S, Pierron G, Ryan KM, Codogno P, Djavaheri-Mergny M (2010). Evidence for the interplay between JNK and p53-DRAM signalling pathways in the regulation of autophagy. Autophagy.

[32] Liu J, Chang F, Li F, Fu H, Wang J, Zhang S (2015). Palmitate promotes autophagy and apoptosis through ROS-dependent JNK and p38 MAPK. Biochemical and biophysical research communications.

[33] Wang D, Sun Q, Wu J, Wang W, Yao G, Li T (2017). A new Prenylated Flavonoid induces G0/G1 arrest and apoptosis through p38/JNK MAPK pathways in Human Hepatocellular Carcinoma cells. Scientific reports.

[34] Taylor CA, Zheng Q, Liu Z, Thompson JE (2013). Role of p38 and JNK MAPK signaling pathways and tumor suppressor p53 on induction of apoptosis in response to Ad-eIF5A1 in A549 lung cancer cells. Molecular cancer.

[35] He Y, She H, Zhang T, Xu H, Cheng L, Yepes M (2018). p38 MAPK inhibits autophagy and promotes microglial inflammatory responses by phosphorylating ULK1. J Cell Biol.

[36] Li Y, Cai X, Guan Y, Wang L, Wang S, Li Y (2016). Adiponectin Upregulates MiR-133a in Cardiac Hypertrophy through AMPK Activation and Reduced ERK1/2 Phosphorylation. PloS one.

[37] Li Y, Ma HL, Han L, Liu WY, Zhao BX, Zhang SL (2013). Novel ferrocenyl derivatives exert anti-cancer effect in human lung cancer cells in vitro via inducing G1-phase arrest and senescence. Acta pharmacologica Sinica.

[38] Huynh KK, Eskelinen EL, Scott CC, Malevanets A, Saftig P, Grinstein S (2007). LAMP proteins are required for fusion of lysosomes with phagosomes. The EMBO journal.

[39] Zaglia T, Milan G, Ruhs A, Franzoso M, Bertaggia E, Pianca N (2014). Atrogin-1 deficiency promotes cardiomyopathy and premature death via impaired autophagy. The Journal of clinical investigation.

[40] Tong M, Saito T, Zhai P, Oka SI, Mizushima W, Nakamura M (2019). Mitophagy Is Essential for Maintaining Cardiac Function During High Fat Diet-Induced Diabetic Cardiomyopathy. Circulation research.

[41] Sendler M, Maertin S, John D, Persike M, Weiss FU, Kruger B (2016). Cathepsin B Activity Initiates Apoptosis via Digestive Protease Activation in Pancreatic Acinar Cells and Experimental Pancreatitis. J Biol Chem.

[42] Zhou J, Tan SH, Nicolas V, Bauvy C, Yang ND, Zhang J (2013). Activation of lysosomal function in the course of autophagy via mTORC1 suppression and autophagosome-lysosome fusion. Cell Res.

[43] Li L, Tan J, Miao Y, Lei P, Zhang Q (2015). ROS and Autophagy: Interactions and Molecular Regulatory Mechanisms. Cell Mol Neurobiol.

[44] He X, Lu J, Dong W, Jiao Z, Zhang C, Yu Y (2017). Prenatal nicotine exposure induces HPA axis-hypersensitivity in offspring rats via the intrauterine programming of up-regulation of hippocampal GAD67. Archives of toxicology.

[45] Benowitz NL, Burbank AD (2016). Cardiovascular toxicity of nicotine: Implications for electronic cigarette use. Trends in cardiovascular medicine.

[46] Andrikopoulos GK, Richter DJ, Dilaveris PE, Pipilis A, Zaharoulis A, Gialafos JE (2001). In-hospital mortality of habitual cigarette smokers after acute myocardial infarction; the "smoker's paradox" in a countrywide study. European heart journal.

[47] Zhang YJ, Iqbal J, van Klaveren D, Campos CM, Holmes DR, Kappetein AP (2015). Smoking is associated with adverse clinical outcomes in patients undergoing revascularization with PCI or CABG: the SYNTAX trial at 5-year follow-up. Journal of the American College of Cardiology.

[48] Li CY, Zhou Q, Yang LC, Chen YH, Hou JW, Guo K (2016). Dual-specificity phosphatase 14 protects the heart from aortic banding-induced cardiac hypertrophy and dysfunction through inactivation of TAK1-P38MAPK/-JNK1/2 signaling pathway. Basic research in cardiology.

[49] Silva RAC, Goncalves AF, Dos Santos PP, Rafacho B, Claro RFT, Minicucci MF (2017). Cardiac Remodeling Induced by All-Trans Retinoic Acid is Detrimental in Normal Rats. Cellular physiology and biochemistry: international journal of experimental cellular physiology, biochemistry, and pharmacology.

[50] Palumbo C, De Luca A, Rosato N, Forgione M, Rotili D, Caccuri AM (2016). c-Jun N-terminal kinase activation by nitrobenzoxadiazoles leads to late-stage autophagy inhibition. Journal of translational medicine.

