# Supplementary material for: Cilostazol alleviate nicotine induced cardiomyocytes hypertrophy through modulation of autophagy by CTSB/ROS/p38MAPK/JNK feedback loop
Source: Int J Biol Sci. 2020 Apr 27;16(11):2001–13. doi: 10.7150/ijbs.43825 (PMC7211170; doi:10.7150/ijbs.43825)
Supplement: Supplementary file 1 — Supplementary figures. [file ijbsv16p2001s1.pdf]

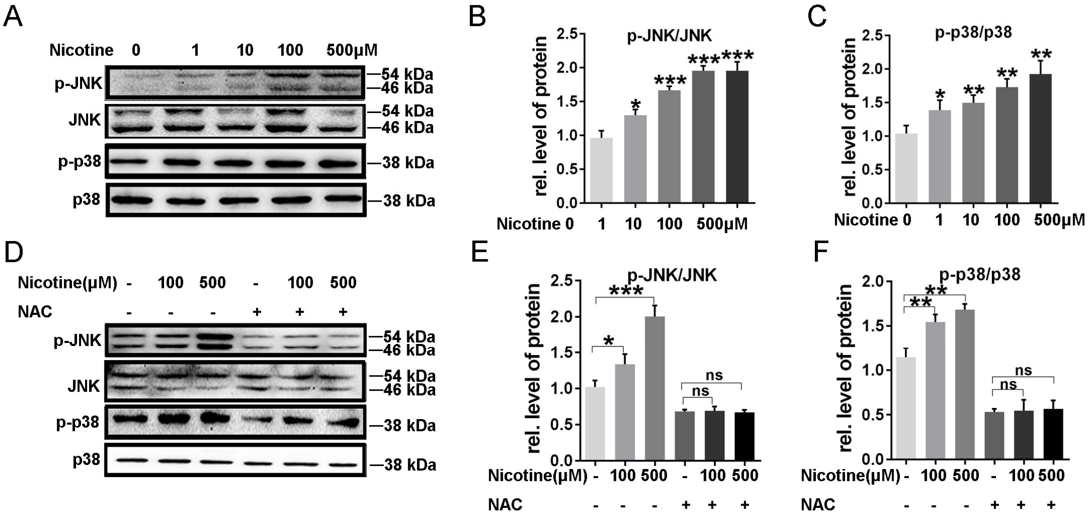

Supplementary Fig. S1 Nicotine activated p38MAPK and JNK signaling in ROS dependent way. Phosphorylation of p38MAPK and JNK levels elevated significantly under stimulation of different concentration of nicotine **(A-C)**, and JNK and p38MAPK activation stimulated by nicotine were inhibited by NAC **(D-F)** (***, $p < 0.001$; **, $p < 0.01$; *, $p < 0.05$, $n = 3$).

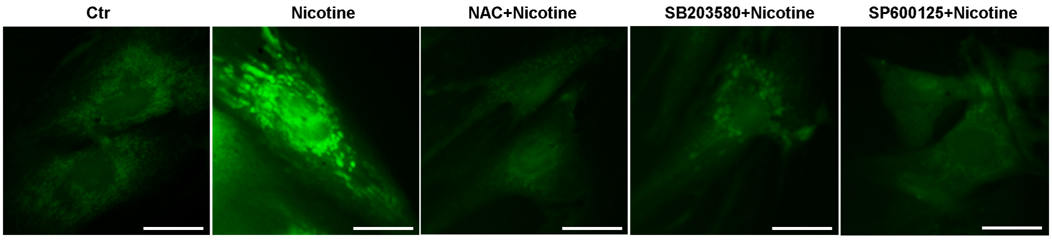

Supplementary Fig. S2 NAC, SB203580 or SP600125 pre-treatment significantly decreased ROS accumulation caused by nicotine. After the pre-treatment of NAC, SB203580 or SP600125, ROS fluorescence intensity decreased remarkably under the stimulation of nicotine. (Scale bar = 20 μm).
